# Effects of beer, wine, and baijiu consumption on non-alcoholic fatty liver disease: Potential implications of the flavor compounds in the alcoholic beverages

**DOI:** 10.3389/fnut.2022.1022977

**Published:** 2023-01-06

**Authors:** Yabin Zhou, Jin Hua, Zhiguo Huang

**Affiliations:** ^1^School of Biological Engineering, Sichuan University of Science and Engineering (SUSE), Zigong, Sichuan, China; ^2^Liquor-Making Biotechnology and Application Key Laboratory of Sichuan Province, Sichuan University of Science and Engineering (SUSE), Zigong, Sichuan, China; ^3^College of Medicine and Public Health, Flinders University, Adelaide, SA, Australia

**Keywords:** non-alcoholic fatty liver disease, flavor compound, beer, wine, baijiu

## Abstract

Non-alcoholic fatty liver disease (NAFLD) is one of the most common causes of chronic liver disease and its global incidence is estimated to be 24%. Beer, wine, and Chinese baijiu have been consumed worldwide including by the NAFLD population. A better understanding of the effects of these alcoholic beverages on NAFLD would potentially improve management of patients with NAFLD and reduce the risks for progression to fibrosis, cirrhosis, and hepatocellular carcinoma. There is evidence suggesting some positive effects, such as the antioxidative effects of bioactive flavor compounds in beer, wine, and baijiu. These effects could potentially counteract the oxidative stress caused by the metabolism of ethanol contained in the beverages. In the current review, the aim is to evaluate and discuss the current human-based and laboratory-based study evidence of effects on hepatic lipid metabolism and NAFLD from ingested ethanol, the polyphenols in beer and wine, and the bioactive flavor compounds in baijiu, and their potential mechanism. It is concluded that for the potential beneficial effects of wine and beer on NAFLD, inconsistence and contrasting data exist suggesting the need for further studies. There is insufficient baijiu specific human-based study for the effects on NAFLD. Although laboratory-based studies on baijiu showed the antioxidative effects of the bioactive flavor compounds on the liver, it remains elusive whether the antioxidative effect from the relatively low abundance of the bioactivate compounds could outweigh the oxidative stress and toxic effects from the ethanol component of the beverages.

## 1. Introduction

Alcoholic beverages not only have been consumed for thousands of years for social, ceremonial, behavioral, and ritual purposes but also are widely consumed. Around 43% of adults (aged 15 years or older) reported consuming alcohol globally in 2016 (1). The average global alcohol consumption was equivalent to approximately 6.43 L of pure ethanol per capita of the adult population in 2015 (2). In 2016, 46% of the total alcohol consumption was beers (34.3%) and wines (11.7%), and 44.8% was spirits (1). South-east Asia region consumed 87.9% of the total spirit globally (1). The major spirit consumed in China (one of the most populated areas of South-east Asia region) is baijiu. Despite its popularity, alcohol consumption ranks as the third most important preventable cause of the disease (3), the fifth-leading risk factor for premature death and disability globally (4), and accounted for 5.1% of the global burden of disease expressed in DALYs (disability-adjusted life years) (1). Excessive alcohol consumption, referring to daily consumption of greater than three drinks (one drink is equivalent to 14 g of pure ethanol), is associated with increased risk of various diseases (5–9), cancers (10–12), and all-cause mortality (13).

On the other hand, however, there are studies demonstrating that low alcohol consumption is not associated with an increased risk of some cancers (14–17). Moreover, some studies from recent years have indicated that low to moderate alcohol consumption, typically 2–3 drinks (approximately 28–42 g of ethanol) per day for men and 1–2 drinks (approximately 14–28 g of ethanol) per day for women, is associated with some beneficial health effects, such as lower risks for cardiovascular disease, dementia, and insulin resistance (18–21). Moderate alcohol consumption is also associated with reduced all-cause mortality (6, 10, 13), and the association is often formed a J-shape relationship (10, 13). Furthermore, some flavor compounds in alcoholic beverages, such as phenolic acids (in beers and wines), organic acids, esters, and terpenoids (22) (in baijiu), may also have additional impacts on health. For instance, the Copenhagen prospective population studies (23) have shown that wine intake is associated with better beneficial effects on all-cause mortality than those from purely alcohol consumption. It was hypothesized that the additional beneficial effects may have come from numerous phenolic compounds present in wine, such as phenolic acids, flavan-3-ols, and anthocyanins.

Non-alcoholic fatty liver disease (NAFLD), one of the most common causes of chronic liver disease worldwide, is clinically diagnosed with the presence of liver fat accumulation ≥5%, determined by radiological imaging techniques, in absence of other known causes (e.g., alcohol, drugs, and virus) (24). The prevalence of NAFLD is increasing constantly, and the current global incidence of NAFLD is estimated to be 24%, with Asia (27%) USA (24%), and Europe (23%) (25). Its prevalence is increasing at a fast pace. In the US alone, for instance, it was projected that the number of patients with NAFLD will increase from 83.1 million (in 2015) to around 100.9 million in 2030 (26). In addition, NAFLD is associated with metabolic syndrome, especially type 2 diabetes and enhances the comorbidities (24, 27). Furthermore, NAFLD, if left unmanaged/poorly managed, can progress to non-alcoholic steatohepatitis (NASH). Approximately 40–50% of the patients with NASH may further progress to hepatic fibrosis, with increased risks of cirrhosis and hepatocellular carcinoma (24). Thus, NAFLD is a growing burden for global healthcare systems. Having good strategies to manage and treat NAFLD, therefore, has become important. The diagnosis of NAFLD reveals NAFLD population consisted of either abstainers or low to moderate alcoholic beverage drinkers. A good understanding of the effects of common alcoholic beverage intake on NAFLD could improve daily NAFLD management and improve the condition of comorbidities.

In this review, the aim is to critically evaluate and discuss the current evidence of effects on liver and NAFLD from human-based and laboratory-based studies on beer, wine, and Chinese baijiu, with a focus on the effects and potential mechanism of ethanol in the beverages, the polyphenols in beer and wine, and the bioactive flavor compounds in baijiu.

## 2. The effects of ethanol on NAFLD

### 2.1. Evidence from studies

**FIGURE 1:**
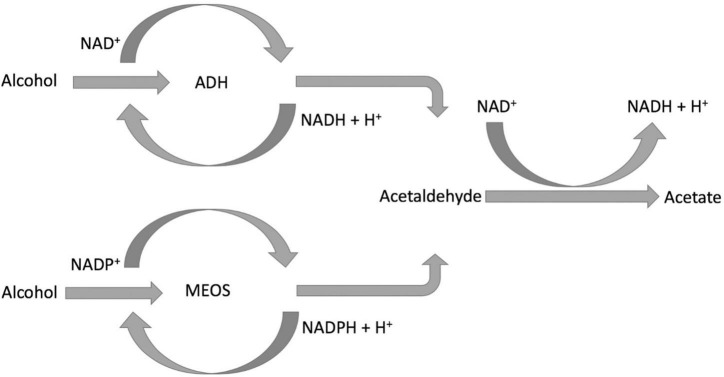
Schematic demonstration of alcohol metabolism.

The consumption of equivalent to 50 g of ethanol per day has an estimated excess risk of 46% for liver cancer, the end stage of NAFLD progression, and the consumption of 100 g of ethanol per day has an excess risk of 66%. A meta-analysis of prospective studies by Turati et al. (12) has shown a positive association between heavy alcohol drinking and liver cancers. Moreover, excessive alcohol consumption is linked to an increased incidence of liver diseases (7). Consumption of alcohol equivalent to 30–50 g of ethanol/day for 5–10 years or longer is associated with an increased risk of alcoholic liver disease (ALD) (5). However, in NAFLD populations, alcohol consumption is either none or low to moderate. The effects of low to moderate alcohol consumption on NAFLD from studies are controversial. On the one side, Bagnardi et al. (28) found daily alcohol consumption no greater than 12.5 g showed no association with liver cancers. In addition, moderate alcohol drinking is associated with a reduced risk for NAFLD and NASH (29, 30). Some studies found low to moderate alcohol consumption could improve serum lipid profiles (31, 32), and alcohol consumption equal to or less than 25 g/day could significantly reduce cardiovascular risk in patients with diabetes (33). Animal studies showed a moderate baijiu (a type of spirit) consumption may potentially improve serum lipid profiles while having no significant damage to the liver (31). On the other side, however, there is evidence suggesting alcohol consumption has no safe limit for NAFLD, even low alcohol consumption could still increase the risk of disease progression to advanced stages (34, 35). The putative mechanism of ethanol’s effects on NAFLD, although not entirely clear, involves the impact of decreased NAD^+^/NADH ratio, oxidation stress, and acetaldehyde on hepatic lipid metabolism. All three factors are the outcome of ethanol’s metabolism in the liver (Figure 1; 36–38).

### 2.2. The mechanisms

After ingestion, approximately 94–98% of the ethanol is removed by two enzyme systems: alcohol dehydrogenase (ADH) (39, 40) and microsomal ethanol-oxidizing system (MEOS) (36, 41). Although ADH is expressed in the cytosol of both gastric mucosa and hepatocytes, the majority is presented in the liver. MEOS is presented predominantly in the endoplasmic reticulum of hepatocytes; it is also expressed in the intestinal mucosa. ADH is metabolizing the majority of the ingested ethanol, especially when consuming no more than three drinks. MEOS, however, plays an important role in removing ethanol after excessive alcohol consumption (such as binge drinking). It is because, in the presence of high blood alcohol concentration (BAC), the activity of MEOS is induced and increased.

After alcohol consumption, gastric ADH eliminates a small fraction of ingested ethanol before it is absorbed and delivered to the liver through the portal vein. The rate of ethanol elimination by Gastric ADH is affected by gender and age, polymorphisms between ethnic groups, rate of drink, and fed or fasted state. This may contribute to the variation and discrepancy of the results in studies. The majority of ingested ethanol is absorbed by the intestinal mucosa and transported to the liver for clearance. The ethanol oxidization catalyzed by ADH also reduces the coenzyme nicotinamide adenine dinucleotide (NAD^+^) to NADH. After heavy drinking, ethanol oxidation by ADH decreases the ratio of NAD^+^/NADH, which could enhance the synthesis of triglyceride and the accumulation of lipids in the liver. For patients with NAFLD, this may potential enhance disease progression. Moreover, the decrease in NAD^+^/NADH ratio also inhibits the oxidation of acetaldehyde, the accumulation of which impairs mitochondria. Mitochondria impairment may lead to lipid accumulation in the liver.

MEOS (42) is predominantly found in the liver, whose main component is cytochrome P450 (CYP) isoform CYP2E1. CYP2E1 oxidizes ethanol to form acetaldehyde and converts nicotinamide adenine dinucleotide phosphate (NADPH) to NADP^+^. MEOS has a higher Michaelis–Menten constant (K_m_) for ethanol than ADH and activates with high BAC. It normally accounts for 20–25% of all alcohol metabolism. Ethanol metabolism facilitated by MEOS also results in the production of various reactive oxygen species (ROS), such as ethoxy radical CH_3_CH_2_O•, hydroxyethyl radical CH_3_C(•)HOH, acetyl radical CH_3_CHO•, and singlet radical ^1^O_2_. After heavy alcohol consumption, the elevated level of ROS generated undergoes covalent bonding to macromolecules on the membrane, subcellular organelles, and subsequently interferes with their biological function (43). In addition, the oxidative stress caused by the ROS on the one hand damages the mitochondria impairing fatty acid beta-oxidation and causing lipid accumulation. On the other hand, oxidative stress on the endoplasmic reticulum (ER) can activate its stress response and enhance fatty acid synthesis.

Acetaldehyde, the direct metabolite of ethanol, is not only IARC classified group 1 carcinogen but also toxic. It is oxidized to acetate by hepatic acetaldehyde dehydrogenase (ALDH), and acetate is further oxidized to CO_2_. The generation of the elevated level of acetaldehyde and/or its slow removal is harmful. The main ALDH isozyme that metabolizes acetaldehyde in the liver is ALDH2. The polymorphism of ALDH2 results in a low-activity enzyme, which has been presented among the East Asian population (such as Han Chinese and Japanese). This polymorphism of ALDH2 may be a potential factor that causes study results discrepancy. Oxidation of acetaldehyde by ALDH requires the reduction of NAD^+^ to NADH. Heavy alcohol consumption increases NADH levels and decreases the NAD^+^/NADH ratio, which could inhibit acetaldehyde oxidation and cause its accumulation. Acetaldehyde can form adducts with DNA, lipids, and proteins; therefore, its accumulation could disrupt normal liver metabolism and may impose a negative impact on the NAFLD population.

## 3. The effects of beer on NAFLD

### 3.1. Evidence from clinical, epidemiological, and laboratory studies

Beer is a type of popular fermented beverage, and its consumption alone took 34.3% of total global alcohol consumption in 2016 (1). Low to moderate beer consumption has been shown to reduce the risk of cardiovascular disease compared to abstainers and heavy drinkers, suggesting the potential cardiovascular protection function of its polyphenols. However, its association with liver function is still inconclusive. The underline mechanism, although unclear, is thought to involve but not limited to the antioxidation, anti-inflammation, and lipid modulation properties of the polyphenolic and bitter acids.

There are limited epidemiological studies investigating beer consumption and liver health, and the outcome remains inconclusive. On the one hand, a positive and significant population-based association between beer consumption and liver disease-led mortality was demonstrated in 221 municipalities in the State of Louisiana in the US (44). In a Danish population–based study, 30,630 men and women with more than five drinks/day of all three types of alcohol were associated with an increased risk for liver cirrhosis compared to abstainers or low alcohol drinkers. However, wine drinkers showed lower risk than beer and spirits drinkers (45). On the other hand, in an Eastern French population study, moderate beer consumption was not associated with increased mortality due to cirrhosis (46).

Various clinical studies and laboratory studies on human subjects have been carried out investigating the beneficial biological properties of polyphenols, bitter acids, and other non-alcoholic components in beer and their potential impact on health. In a randomized crossover trial involving 11 healthy middle-aged non-smoking men, beer consumption (equivalent to 40 g of ethanol per day) for 3 weeks did not increase the values of liver enzymes: gamma-glutamyltransferase (GGT), aspartate aminotransferase (AAT), and alanine aminotransferase (ALT) (47). Similar results were obtained from another crossover trial involving 60 healthy Spanish adults (31 men and 29 women), in which the levels of hepatic enzymes (GGT, GOT, and GPT) are unchanged after beer consumption (equivalent to 11 g/day for women and 22 g/day for men) for 1 month (48). However, in the crossover trial involving 10 middle-aged men and 10 post-menopausal women, the levels of both GGT and ALT showed a slight increase (but still within the normal clinical range) after beer consumption (equivalent to 40 g/day for men and 30 g/day for women) for 3 weeks. But interestingly, inflammation markers, C-reactive protein, and fibrinogen were decreased significantly, indicating anti-inflammatory action, after the 3-week beer consumption (49).

In a laboratory study, antioxidant melatonin was detected and measured in 18 brands of beer with different alcohol concentrations (50). In addition, serum samples from seven healthy human subjects were analyzed before and after beer consumption, which showed both melatonin and total antioxidant status increased after beer consumption. This suggests beer consumption may increase the antioxidative capability of human serum attributed to melatonin and other compounds beer contains. This coincides with the results from other studies, which found increased plasma polyphenolic contents and antioxidant capability (51, 52).

*In vitro* and *in vivo* studies also showed the beneficial biological effects of polyphenols, bitter acids, and other non-alcoholic components in beer. In a study using an aluminum-induced neurotoxicity murine model, the beer treatment group showed significant lower lipid peroxidation, higher expression of antioxidant enzymes (at mRNA levels), and lower expression (mRNA) of inflammation marker TNFα (53). The authors speculated polyphenols (such as resveratrol) and antioxidants (such as folic acid) in beer may have contributed a part to the antioxidation and anti-inflammation properties of the beer. These studies showed that beer-derived polyphenols may be absorbed and reach the blood circulation to exert biological functions. Two recent studies by Shafreen et al. and Tung et al. further demonstrate that serum polyphenols (come from beer) can bind and interact with serum proteins, such as human serum albumin, plasma circulation fibrinogen, and low-density lipoprotein to exert antioxidant functions (54, 55). In an *in vitro* study on peripheral blood mononuclear cells by Winkler et al., beer components were shown to increase neopterin production and tryptophan degradation and reduce ROS generation by inhibiting the production of pro-inflammatory cytokine interferon-γ (56).

### 3.2. The effects and putative mechanisms of the main flavor compounds in beer

Beer is fermented from cereals and hops (Humulus lupulus), consisting of over 90% of water, carbohydrates, ethanol, (more than 50) polyphenolic compounds, bitter acids (e.g., humulones and lupulones), proteins, B-complex vitamins, and trace amounts of minerals (57, 58). The alcohol concentration of beer varied approximately from 3.5 to 10% (w/v) in different kinds of beers. The main non-alcoholic flavor compounds of beer thought to exert beneficial biological functions are (1) polyphenolic compounds: xanthohumol (around 0.2 mg/L), isoxanthohumol (around 0.6–3.4 mg/L), and phenolic acids (25–29 mg/L); (2) bitter acids: humulones (approximately up to 4 mg/L), lupulones (around 0.012–0.14 mg/L), and iso-humulones (around 10–100 mg/L) (57).

Among the non-alcoholic compounds in beer, the hop-derived phenolic compounds and bitter acids have been shown to modulate hepatic lipid metabolism and process anti-inflammatory, antioxidative, and anticarcinogenesis properties (Table 1). Xanthohumol, of which beer is the main human diet source, is a bioactive multifunctional prenylated flavonoid from the female inflorescence of the hop plant (59, 60). It has been shown with the capability to modulate hepatic lipid metabolism (61, 62). In type 1 diabetic rodent model, insulin deprivation led to down-regulation of fatty acid synthase (FAS), inactivation of Acetyl-CoA Carboxylase (ACC), and inhibition of lipogenesis (61). Xanthohumol was able to activate ACC, increase the expression of FAS, and restore some proportion of lipogenesis, through a mechanism not clearly understood. In mice fed with a high-fat diet, xanthohumol was able to reduce triglycerides and cholesterol content in the liver and skeletal muscle by inhibiting lipogenesis and lipid uptake and promoting β-oxidation (62). The putative mechanism involves xanthohumol activation of AMP-activated protein kinase (AMPK), which then inhibits the expression of sterol regulatory element-binding protein 1c (SREBP-1c), downstream ACC and FAS, down-regulation of the expression of lipid transporter CD36. In addition, xanthohumol was shown to inhibit liver fibrosis in type 1 diabetic rodent model (61). The mechanism, although not clear, is speculated to involve anti-inflammation and antioxidation actions as demonstrated in another study based on the same type 1 diabetic rodent model (63). Furthermore, xanthohumol was shown in a rodent model to protect the liver and the colon from DNA damage, and preneoplastic lesion caused by cooked food mutagen (64), indicating the capability to prevent liver cancer development from more general carcinogens, such as ethanol and acetaldehyde. Xanthohumol can be converted to isoxanthohumol during the brewing process and/or in the stomach. As one of the major flavonoids in normal beers, isoxanthohumol may also involve in modulating hepatic lipid metabolism, anti-inflammation, and antitumor (57, 61). Isoxanthohumol can be further converted to 8-prenylnaringenin by the microbiota in the intestine (57). 8-Prenylnaringenin not only exerts hormonal function as the strongest phytoestrogen but also involves modulating lipid metabolism (62, 65). Landmann et al. showed that normal beer (brewed with the hop) was able to attenuate hepatic lipid accumulation in a binge-drinking mouse model (66). The putative mechanism is shown to be the inhibition of hepatic iNOS induction and lipid peroxidation. The further study by the same group showed that iNOS and lipid peroxidation inhibition may be exerted by iso- α-acids (iso- humulones) from hop (67). The authors speculated the protection effects may also involve other compounds in the hop extracts, such as β-acids (lupulones).

**TABLE 1:** Bioactive flavor compounds in beer and their beneficial effects.

Compounds	Demonstrated beneficial effects	References
Xanthohumol	Modulate lipid metabolism; antioxidation; anti-inflammation; anticarcenogenesis	(59–64)
Isoxanthohumol	Modulate lipid metabolism; anti-inflammation; antitumor	(57, 61)
8-Prenylnaringenin	Hormonal function (Phytoesrongen); modulate lipid metabolism	(62, 65)
Bitter acids	Antioxidation; modulate lipid metabolism	(66, 67)
Other polyphenolic compounds	Antioxidation; anti-inflammation	(47–52)

**FIGURE 2:**
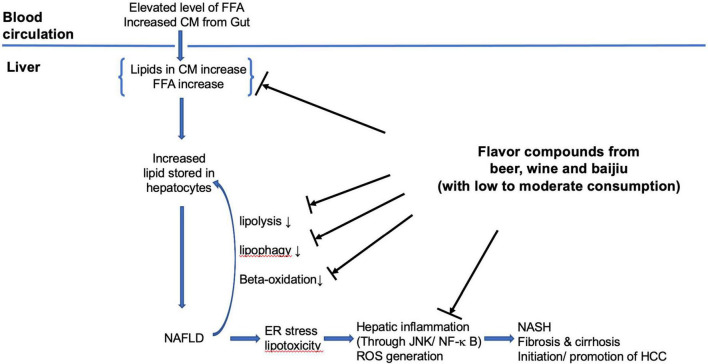
The putative mechanisms of flavor compounds on NAFLD. FFA, free fatty acids; CM, chylomicron remnants; ER, endoplasmic reticulum; HCC, hepatocellular carcinoma.

The putative mechanisms that beer flavor compounds involved can be summarized as following three main areas (refer to Figure 2). (1) Modulate hepatic lipid metabolism: down-regulating hepatic lipogenesis, reducing hepatic lipid uptake from circulation, and enhancing β-oxidation; (2) antioxidation: as antioxidant removing ROS, increasing quantity and activity of antioxidant enzymes, and inhibition of lipid peroxidation; (3) anti-inflammation: preventing hepatic inflammation (through JNK/NF-κ B) caused by lipid accumulation induced endoplasmic reticulum stress in hepatocyte and lipid peroxidation. The exact process and how these are integrated remain elusive.

## 4. The effects of wine on NAFLD

### 4.1. Evidence from clinical and epidemiological studies

Wine is a type of popular alcoholic beverage fermented from grape vines. The term French paradox describes the observation of a lower incidence of coronary heart disease in France than in other Western countries, despite similar intake of high levels of saturated fat (68). This was based on epidemiological studies, which suggested the observation was attributed to the beneficial effects of red wine consumption, on the data collected from the MONICA project organized by WHO. Since then, epidemiological studies and human trials have been carried out investigating the potential health benefit of wine. Among them, only limited studies directly investigated the effects of wine on NAFLD prevalence and progression with promising outcomes; however, more data are needed before any conclusion can be drawn. Dunn et al. performed the first epidemiological study investigating the association between modest consumption of wine and NAFLD (69). The study showed a lower NAFLD prevalence in participants who consumed up to four ounces of wine daily when compared to abstainers and participants whose daily consumption of up to 12 ounces of beer, 1 ounce of liquor, or 1 drink of mixed alcoholic drinks. The wine drinkers also demonstrated a lower prevalence of diabetics and other metabolic syndrome features in the study. However, this study did not demonstrate the safety of modest wine drinking in patients with NAFLD. A single-center cohort study by Mitchell et al. showed modest wine consumption (<70 g of ethanol per week without binge consumption) was associated with a significantly lower risk of advanced hepatic fibrosis compared to abstinence among patients with NAFLD (70). Some studies investigated the association between wine consumption and the risk of liver cirrhosis, the effects of wine drinking on hepatic lipid levels, functions, serum cholesterol profiles, and NAFLD co-exist metabolic syndrome. The outcome of the studies was inconsistent and inconclusive. In a prospective study in the Copenhagen area, Becker et al. found an increase in the risk of liver cirrhosis with increasing total alcohol intake for beer, wine, and spirit, but wine consumption showed a lower risk (45). In a large cohort prospective study including 1.3 million middle age UK women, with a mean of 15 years of following up of 401,806 women, the authors found that the risk of liver cirrhosis increased with the total amount of alcohol intake (event with moderate consumption), the increase of risk, in a given weekly intake of alcohol, was also associated with consumption without a meal or daily consumption, regardless whether drinking only wine or more than one type of alcoholic beverages (71). A randomized crossover trial by Beulens et al. showed 4 weeks of red wine consumption (40 g of ethanol per day) did not significantly increase liver fat compared to 4 weeks of consumption of de-alcoholized red wine (72). An interventional cohort study by Rajdl et al. showed, although there was an increase in liver enzymes AST (within the normal reference range) and ALT (slightly exceeded upper threshold), white wine consumption is associated with an increase in antioxidative effects (73). In a prospective randomized trial involving 44 healthy subjects (32 women and 12 men), Kechagias et al. showed an increase in ALT and AST (within an upper reference threshold), decrease in LDL cholesterol, and a trend of hepatic triglyceride content increase for subjects with moderate red wine consumption for 90 days (74). These changes in the red wine consumption group were significantly different when compared with the alcohol abstention group. Taborsky et al. carried out the prospective, multi-center, randomized In Vino Veritas study comparing the effects on healthy subjects between red and white wine consumption (75). The results showed that the changes in total cholesterol, HDL, LDL, triglyceride, liver function, and other markers during the 12-month wine consumption were not varied significantly between red and white wine groups, regardless of the significant difference in the polyphenolic compounds between the two wines. However, when comparing the baseline within each group, both groups showed a significant reduction in LDL for time points at 6 months and 12 months, a significant total cholesterol reduction at 6 months, the red wine group showed a significant HDL reduction at 6 months and a significant total cholesterol reduction at 12 months. Type 2 diabetes is one of the most common metabolic syndromes that co-exist with NAFLD (24). In a 2-year randomized intervention trial, Gepner et al. demonstrated that red wine consumption significantly increased HDL-C levels and decreased the total cholesterol/HDL-C ratio (76). When compared to the non-drinking (water) group, the overall value of metabolic syndrome components was further significantly decreased in the red wine group.

### 4.2. The effects and putative mechanisms of the phenolic compounds in wine

Wine contains water, carbohydrates, organic acids, alcohol, polyphenols, minerals, and B vitamins. The rich phenolic compounds of wine (especially red wine) are thought to provide potential health benefit effects (Table 2). The main phenolic compounds in wine are stilbenes (resveratrol), phenolic acids, and flavonoids (flavan-3-ols, Anthocyanins, quercetin) (77–80). Although the mean level of resveratrol (a type of stilbenes) is 7 mg/L in red wine, the total stilbenes level could be up to 20 mg/L (80, 81). The levels of catechin and epicatechin, as main flavan-3-ols, are approximately 100 and 75 mg/L, respectively. The amount of anthocyanins and quercetin is up to 500 mg/L and around 16 mg/L, respectively (80, 81). Various mechanisms (concerning the polyphenolic compounds in wine) have been proposed for the potential beneficial effects of wine, especially red wine on liver metabolism and NAFLD. These include the antioxidation effects, anti-inflammatory effects, and modulation of lipid metabolism. Resveratrol, one of the most important phenolic compounds in wine, demonstrated the capability to ameliorate antioxidative stress and inflammation and modulated hepatic lipid metabolism (82, 83). Resveratrol is not only an antioxidant, scavenging ROS, HO, peroxyl radicals, and chelating metal ions interacting with ROS (84–86), but also capable of increasing the activity of hepatic antioxidation enzymes, such as superoxide dismutase, catalase, and glutathione peroxidase (87–90). Resveratrol has also been shown to be able to modulate hepatic lipid metabolism by activation of sirtuin 1 (SIRT 1)–AMPK signaling, which on the one hand promotes the fatty acid beta-oxidation by activating peroxisome proliferator-activated receptor α (PPARα), PPARγ co-activator 1α (PGC1α), and their target genes, on the other hand, down-regulates fatty acid synthesis through SREBP-1c inhibition (91). Additionally, resveratrol was shown to reduce intracellular lipid droplets possibly by promoting autophagy in HepG2 cells (92). Resveratrol has also been shown in studies to possess anti-inflammatory properties, such as inhibiting infiltration of macrophage and recruitment of Kupffer cells, reducing TNFα levels (93–95).

**TABLE 2:** Bioactive flavor compounds in wine and their beneficial effects.

Compounds	Demonstrated beneficial effects	References
Resveratrol	Antioxidation; anti-inflammation; reduce lipid accumulation	(82–95)
Quercetin	Antioxidation; anti-inflammation; antiapoptosis; hepatoprotective	(77, 78, 80)
Anthocyanins	Antioxidation; anticancer	(79, 80)
Total phenolic compounds	Antioxidation; anti-inflammation; modulate lipid metabolism; antifibrosis; improve serum lipid profile; improve metabolic syndrome condition	(68–76, 80)

The putative mechanisms that phenolic compounds in wine, such as resveratrol involved in can be summarized in three main areas (refer to Figure 2). (1) Antioxidation: as antioxidants scavenging ROS, HO, and peroxyl radicals, increasing quantity and activity of antioxidant enzymes and inhibition of lipid peroxidation; (2) modulating hepatic lipid metabolism: activation of SIRT 1–AMPK signaling leading to inhibition of hepatic lipogenesis and enhancing β-oxidation, promoting lipid autophagy; (3) anti-inflammation: through inhibition of NF-κ B pathways. The exact processing involved is not entirely clear, more studies are needed.

## 5. The effects of baijiu on NAFLD

### 5.1. Evidence from laboratory studies

**FIGURE 3:**
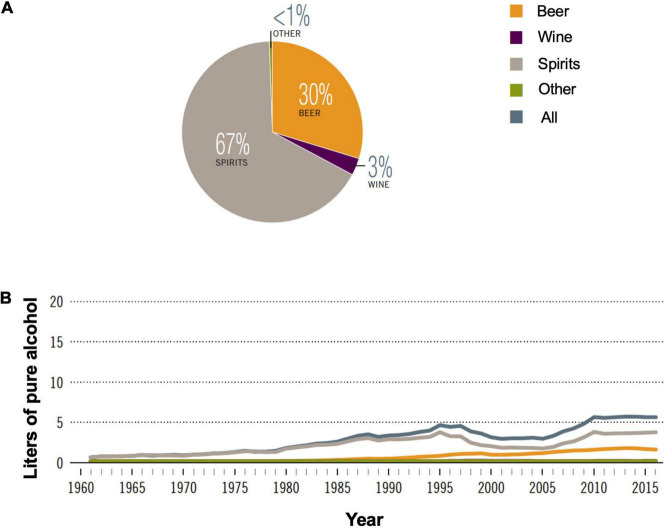
Alcohol consumption in China. **(A)** Proportion of different alcoholic beverages consumed in 2016; **(B)** recorded alcohol consumption per capita in adults (age > 15 years), 1961–2016. Adapted with permission from ref (1) under Creative Commons Attribution-Non-Commercial-ShareAlike 3.0 IGO license. (CC BY-NC SA 3.0 IGO; https://creativecommons.org/licenses/by-nc-sa/3.0/igo).

Among the alcoholic beverages consumed globally, 44.8% are spirits. In China, alcohol consumption has been increasing since the 1960s with total recorded consumption reaching equivalent to 5.7 L of pure alcohol per capita in 2016 (Figure 3A). Spirits consumption is 67% of all alcoholic beverages in 2016 (Figure 3B; 1). Baijiu, the main category of spirits consumed in China, is produced by unique multi-strain and solid-state fermentation techniques.

A study (96) based on the human liver cell line, Hep3B, has shown a non-alcoholic residue of Maotai (a brand of baijiu), is able to up-regulate GST A1, an antioxidant-responsive element. This subsequently promotes antioxidative activity through an ERKs- and p38 K-dependent pathway, which may reduce oxidative stress caused by alcohol metabolism and provide protection to the liver. Subsequent animal studies (97, 98) on Maotai have shown that it has different effects on the liver than that of the same amount of alcohol. It significantly induced various antioxidation factors, heme oxygenase-l, metallothionein, Nrf2, and GCLC. A tetrapeptide from sesame flavor-type baijiu has been shown to promote hepatic antioxidation factors through various mechanisms to counteract the oxidative stress caused by alcohol metabolisms (99). A recent animal study (100) compared the effects of daily consumption of equivalent to approximately three drinks of baijiu or an equivalent amount of pure alcohol solution. Results showed that the baijiu treatment group has significantly less liver injury and steatosis. Further study on approximately 1.5 drinks of baijiu or equivalent pure alcohol solution showed pure alcohol solution treatment group induced significantly higher plasma ALT and hepatic triglyceride levels. The non-alcoholic flavor compounds in baijiu have been shown in an animal study, to be able to attenuate liver damage caused by ethanol potentially through differential impact on host gut microbiota (101).

Animal and *in vitro* studies have shown baijiu posts less injury to the liver than an equivalent amount of pure alcohol. This coincides with the speculation that the non-alcoholic components, especially biologically active compounds of baijiu may have additional effects on the liver that is apart from alcohol. However, due to the limited evidence, this hypothesis is still controversial. First, more laboratory studies are needed to demonstrate what are the main compounds that could convey these effects (either beneficial or harmful), the mechanisms, and potential interactions between the compounds and ethanol. Second, evidence is needed to show that the amount of compounds in baijiu is sufficient to convey the proposed effects. Moreover, the evidence from the epidemiology and clinical studies is insufficient and inconclusive (102, 103). Better controlled epidemiology and clinical studies, such as randomized controlled trials, would be needed (104).

### 5.2. The effects and putative mechanisms of the main flavor compounds in baijiu

It is proposed that the non-alcoholic components, such as polyphenols in alcoholic beverages, may have additional beneficial effects (83, 105). Baijiu, a type of distilled spirit produced through solid-state fermentation (106), has contained more than 1,874 kinds of identified flavor compounds (22, 106). Among these compounds, there are at least 138 kinds have been shown to be bioactive (22). It is speculated that the biologically active compounds in baijiu may have some protective effects on the liver from the injury caused by ethanol metabolism when baijiu consumption is low to moderate (Table 3; 22, 100).

**TABLE 3:** Bioactive flavor compounds in baijiu and their beneficial effects.

Compounds	Demonstrated beneficial effects	References
Ferulic acid (phenolic acid)	Antioxidation; anti-inflammation; liver protective;	(81, 107)
Acetic acid, butyric acid, linoleic acid, alpha-linolenic acid, lactic acid and L-Malic acid (organic acids)	Antibacterial; serum cholesterol and triglycerides reduction; anti-inflammation; Antioxidation; antiapoptosis; hepatoprotective	(110–114)
Tetramethylpyrazine (pyrazines)	Hepatoprotective; Antioxidation	(115, 116)
Ethyl linolenate and ethyl linoleate (ethyl esters)	Improve serum lipid profile	(119)
Terpenes	Antioxidation; antibacterial; potential hepatoprotective	(120)
5-hydroxymethyl furfural	Anti-inflammation; serum cholesterol reduction; antitumor	(121–123)
Lichenysin; tripeptide Pro-His-Pro	Antibacterial; antiviral; Antioxidation	(125–127)

The biological active volatile compounds include phenols, organic acids, esters, terpenes, pyrazines, sulfur compounds, and furan derivants. The non-volatile compounds include polyols, peptides, amino acids, vitamins, and minerals. Various phenols have been identified by techniques, such as GC-MS, HPLC-MS, and GC-TOF-MS. Five of them are shown to have beneficial effects. Ferulic acid, the main ingredient of several Chinese herbals, has been shown to have antioxidation and anti-inflammation effects and may be protective of the liver against the oxidative stress caused by alcohol metabolism (81, 107). Baijiu contains 127 organic acids, which are important to the baijiu flavor. In the sesame-aroma type of baijiu, there are eight acids that have a quantity higher than 10 mg/L (108). In baijiu, Luzhoulaojiao, the quantity of the acid reaches as high as 300 mg/L (Table 4; 109). The acids may potentially have beneficial effects. To date, 16 acids have been reported to be health beneficial. Acetic acid, butyric acid, linoleic acid, alpha-linolenic acid, lactic acid, tartaric acid, and L-Malic acid are typical ones (110–114). The non-saturated acids, such as linoleic and linolenic acids, may improve lipid profiles. The acid may also help regulate liver lipid synthesis. SCFA, such as butyric acid (around 80 mg/L in baijiu), are known to involve in the regulation of energy homeostasis, obesity, immune system, brain function, and colorectal cancer prevention (113). Although the concentration of butyric acid in baijiu is low, it may serve as a source of dietary intake of butyrate to maintain its physiological concentration in the human body.

**TABLE 4:** Abundant and bioactive compound in Luzhoulaojiao.

Compounds	Concentration (mg/L)
Ethyl hexanoate	2221 @ 12
Ethyl acetate	693 @ 8
Ethyl lactate	316 @ 4
Hexanoic acid	300 @ 111
Butanoic acid	109.7 @ 0.7
Ethyl butyrate	46.3 @ 0.8
Heptanoic acid	36 @ 1
Furfural	30.96 @ 0.05
Ethyl valerate	10.7 @ 0.1
Phenylethyl Alcohol	3.66 @ 0.03
Ethyl heptanoate	3.38 @ 0.04
1-Hexanol	2.76 @ 0.02
1-Butanol	1.784 @ 0.005

Adapted with permission from ref (109) under a Creative Commons Attribution 4.0 International License.

Pyrazines are a category of biologically active compounds in baijiu and may have health benefits. A pyrazine and its several derivants, such as tetramethylpyrazine, have been detected in Maotai, Laobaigan, Yanghe River Daqu, and Fenjiu. Tetramethylpyrazine is the main active ingredient of Rhizoma Ligustici Chuanxiong, a Chinese herbal that has long been used to treat liver disease and protect the liver from fibrosis (115, 116). It has antioxidation properties and enhances triglyceride degradation. Esters are the major flavor compounds in baijiu. To date, 510 esters have been identified in baijiu (117). For instance, lactic acid ethyl ester is approximately 900 mg/L in soy sauce aroma type baijiu (118). At least five of them have been reported to have beneficial effects. Fatty acid esters, such as ethyl linolenate and ethyl linoleate may have a regulatory property on cholesterol synthesis (119). Alpha-angelica lactone, an important ingredient of Chinese herbal Angelicae sinensis radix and Rhizoma Ligustici Chuanxiong, has been detected in Jiannanchun and Gujinggong. It is shown to protect and regulate the immune system, especially speeding the immune system recovery after chemotherapy. It may potentially regulate liver immune response upon oxidative stress caused by alcohol metabolism and prevent the progression of ALD. Terpenes are a category of important compounds in baijiu. Fifty-two of the seventy-six identified terpenes in baijiu have been reported to be health beneficial (106). They process antioxidation, antiviral, and antibacterial properties, which may potentially be liver protective (120). Its concentration in baijiu can be as high as 3,400–3,600 μg/L. Baijiu also contains sulfur compounds, to date, 73 compounds have been identified. At least six of the identified sulfur compounds have been reported to be beneficial to health. One of their properties is antioxidation, which protects cells from injury from oxidative stress. Furans have also been identified in baijiu, which have antitumor properties. In particular, 5-hydroxymethyl furfural has been shown to inhibit tumor progression and anti-inflammation and is capable to reduce the serum cholesterol level (121–123). Among the non-volatile compounds, peptides are recently identified as bioactive compounds in baijiu. Lichenysin is a lipopeptide identified in Dongjiu (124) with a concentration as high as 112 μg/L. One of its properties is antibacterial activity and antiviral activity (125, 126). Based on structural similarity to surfactin, it is speculated that lichenysin may process antitumor properties through tumor cell G2/M arrest; however, experimental confirmation is needed. A tripeptide Pro-His-Pro (PHP) has been identified in the sesame-aroma type of baijiu Gujinggong (127). An *in vitro* study on human liver cell line HepG2 cells has demonstrated its ability to up-regulate cellular antioxidation enzymes, such as superoxide dismutase, catalase, and glutathione peroxidase through Nrf2/antioxidant response in the element signaling pathway. Therefore, PHP pre-treatment was able to prevent HepG2 cells from oxidative stress induced by 2,20-azobis (2-methylpropanimidamidine) dihydrochloride.

The proposed mechanisms of the flavor compounds in baijiu involved can be summarized as antioxidation, anti-inflammation, and lipid metabolism modulation (refer to Figure 2). For antioxidation mechanisms, the flavor compounds can act as antioxidants to remove ROS and to up-regulate cellular antioxidation enzyme quantity and activities. For the anti-inflammatory effects, the flavor compounds may alleviate hepatic inflammation by modulating NF-κB-regulated pathways. They can also reduce the hepatic inflammation induced by liver cell endoplasmic reticulum stress caused by intracellular oxidation stress (through antioxidation pathways). For hepatic lipid metabolism modulation, the flavor compounds enhance lipid degradation and β-oxidation and at the same time reduce lipogenesis.

Although the aforementioned compounds in baijiu have potential biological effects, many of them are in low concentrations. For moderate consumption of baijiu, therefore, it is less likely that the intake of each of those low-concentration compounds reaches a sufficient level to exert any effects. Therefore, it is important to identify the main compounds that possess the beneficial effects and potential combinational effects they impose as a whole and elucidate the mechanism underlining the combinational effects.

## 6. Discussion

It is commonly accepted that excessive alcohol consumption or binge drinking (128) leads to ALD as well as advanced stages of NAFLD. For low to moderate alcohol consumption, controversial evidence exists on ethanol effects on NAFLD from epidemiology and clinical studies, and the mechanism is not entirely clear. The genetic variance of ADH and ALDH among the study population could convey variation in effects on the liver.

In addition, the findings from epidemiology and clinical studies on the effect of the polyphenolic compounds from beer and wine on NAFLD are inconsistent and inconclusive. For epidemiological studies, for instance, there are variations in a range of factors that contribute to the resulting inconsistency. These factors include but are not limited to the drinking patterns (frequency and amount, binge drinking or not, with/without a meal, proportion of wine among total alcohol consumed), variation of wine consumed, biological variation of investigated subjects (ethnic, gender, age, health status, etc.), study duration, and population. Hence, more well-controlled, long-term, randomized trials are needed.

For baijiu, there are very limited epidemiology and clinical studies available, most of the evidence is from laboratory based *in vitro* and *in vivo* studies. Different from beer and wine, baijiu contains a much higher concentration of alcohol. One could speculate low to moderate baijiu consumption could result in a much lower intake of bio-activate non-alcoholic flavor compounds for any potential beneficial effects. However, laboratory studies demonstrate the significant effects between baijiu and pure ethanol (100, 102). More laboratory studies are needed to first verify this difference and then elucidate the reason/mechanisms behind it. In addition, more specifically designed, baijiu based epidemiology, and clinical studies are needed to further investigate the effects of baijiu on the liver and the mechanism.

## 7. Conclusion

The non-alcoholic bioactive flavor compounds in beer, wine, and baijiu have been shown beneficial to NAFLD. The underline mechanism for the beneficial effects is proposed to involve modulation of lipid metabolism, reduction of oxidative stress and damages, and alleviation of inflammation. However, it is inconclusive whether low to moderate consumption of these three types of beverages is beneficial to NAFLD. For patients with NAFLD, it is recommended to abstain, although a low level of alcohol consumption may be alright. For normal people, the recommendation is either abstaining or consuming low to moderate alcohol without binge drinking.

## Author contributions

YZ: conceptualization and drafting of the manuscript. JH: manuscript writing. ZH: conceptualization, manuscript draft review, and modification. All authors contributed to the article and approved the submitted version.
